# The structure, role, and procedures of Korean expert committee on immunization practices

**DOI:** 10.1016/j.jvacx.2024.100601

**Published:** 2024-12-10

**Authors:** So Yun Lim, Han Wool Kim, Young June Choe, Bin Ahn, Hyun Mi Kang, Jiyoung Park, Geun Yong Kwon, Seung Ho Lee, Sunghee Kwon, Eun Hwa Choi

**Affiliations:** a Department of Infectious Diseases, Asan Medical Center, University of Ulsan College of Medicine, Seoul, Republic of Korea; b Department of Pediatrics, Hallym University Sacred Heart Hospital, Anyang, Republic of Korea; c Department of Pediatrics, Korea University College of Medicine and Korea University Anam Hospital, Seoul, Republic of Korea; d Department of Pediatrics, College of Medicine, The Catholic University of Korea, Seoul, Republic of Korea; e Vaccine Bio Research Institute, College of Medicine, The Catholic University of Korea, Seoul, Republic of Korea; f Department of Pediatrics, Korea University Ansan Hospital; g Division of Immunization Planning, Korea Disease Control and Prevention Agency, Cheongju, Republic of Korea; h Department of Pediatrics, Seoul National University College of Medicine, Republic of Korea; i Department of Pediatrics, Division of Pediatric Infectious Diseases, Seoul National University Children's Hospital, Seoul, Republic of Korea

**Keywords:** Korea expert committee on immunization practices, Immunization policy, National vaccination program

## Abstract

Vaccination is a cornerstone of public health, preventing infectious diseases with significant contribution to human health. In South Korea, the Korea Expert Committee on Immunization Practices (KECIP) plays a pivotal role in guiding national vaccination policies. In this comprehensive review, we investigated the history, legal basis, operation, and achievements of the KECIP, highlighting its critical role in shaping the country's successful vaccination program.

We analyze the KECIP's diverse responsibilities, including deliberating on national immunization programs, establishing vaccination criteria, managing targeted infectious diseases, and formulating eradication strategies. Also, we revealed its well-defined structure, specialized subcommittees, and ethical protocols that ensure transparency and integrity. Furthermore, we explored the KECIP's strategic evolution, showcasing its contributions to expanding vaccine coverage, implementing emergency approvals, and optimizing foundational vaccinations for all age groups as well as special populations including immunocompromised individuals.

By combining scientific rigor, expert insights, and a commitment to public health, the KECIP had navigated the dynamic landscape of infectious disease control, contributing significantly to South Korea's impressive vaccination achievements.

## 1 Introduction

Vaccination is the most effective and cost-benefit efficient means of preventing infectious diseases. [1,2] In the case of smallpox, it has already been eradicated from the global landscape, [3] and many other diseases preventable by vaccines have seen a significant reduction in occurrences, contributing to the enhancement of human health. [4] Vaccination also reduces the economic burden of diseases, protecting citizens from unnecessary suffering caused by preventable illnesses and ultimately improving the quality of life. [5]

Given these contributions, vaccination is recognized as a crucial aspect of public health policies worldwide. Countries worldwide promote government-recommended national immunization programs to maintain a specified level of vaccination coverage. [6] Recently, the global threats of diseases such as smallpox [3,7], and avian influenza [8] have underscored the importance of vaccine stockpiling and procurement from a national security perspective. [[9], [10], [11], [12], [13]]

However, the establishment and execution of such vaccination policies require a systematic and comprehensive approach. The Korea Expert Committee on Immunization Practices (KECIP) is responsible for developing evidence-based recommendations on immunization policies and practices, including vaccine schedules and target populations. KECIP also develops and operates systematic frameworks for evidence review, decision-making, and communication of recommendations. Other departments within the Korea Disease Control and Prevention Agency (KDCA) are tasked with implementation activities such as the production, procurement, and distribution of vaccines, provision of facilities and equipment, and funding for vaccination programs. To effectively implement vaccination policies, comprehensive strategies are essential across various operational and surveillance fields. While KECIP focuses on developing recommendations and maintaining decision-making processes, other KDCA departments execute operational logistics and establish robust surveillance mechanisms to monitor vaccination coverage and the incidence of targeted diseases.

In this context, this review provides an overview of the history of technical advisory activities in South Korea, the current status, and policy directions for each stage of vaccination. The KECIP is a specialized committee that deliberates and decides on the criteria and methods for immunization, designation or cancellation of infectious diseases subject to vaccination, and eradication plans for the management of infectious diseases targeted for vaccination.

## 2 History of Korea Expert Committee on Immunization Practices

The inception of Korea Expert Committee on Immunization Practices dates back to June 8, 1992, established in alignment with the Ministry of Health and Welfare Order No. 641. Its legal standing was further solidified in August 1994 when it transitioned into a legal entity under the nomenclature of the Immunization Review Committee, as per Article 10–2 of the Infectious Diseases Prevention Act, later renamed as the Infectious Disease Control and Prevention Act. [14] Commencing its deliberations in February 1995, the committee has convened multiple sessions annually. The committee follows a biannual schedule for regular meetings, with additional sessions held as needed. In March 2011, during that year's eighth session, the committee underwent a nomenclatural shift to become the Korea Expert Committee on Immunization Practices (KECIP). The committee continues this meeting structure, with session numbers resetting each calendar year. As of August 2023, the seventh session of the KECIP for that year was operational, reflecting the committee's continuous engagement in immunization practices throughout the year. [15] The Korea Disease Control and Prevention Agency (KDCA) established the Infectious Disease Control Committee as a pivotal body for deliberating on major policies concerning infectious disease prevention and management. Within this structure, the KECIP was positioned as a specialized subcommittee, focusing on immunization policies and practices. This organizational arrangement allows KECIP to function as South Korea's National Immunization Technical Advisory Group (NITAG).

## 3 Legal Basis

**Fig. 1:**
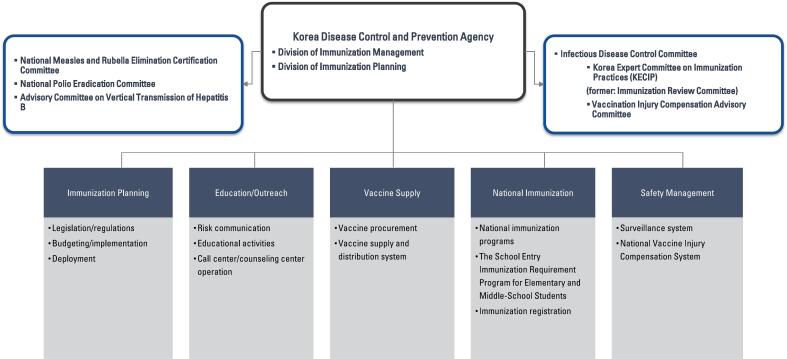
Organization and Roles of the Korea Disease Control and Prevention Agency for Immunization Policy.

The organizational structure and roles of the Korea Disease Control and Prevention Agency (KDCA) specifically in the context of immunization policy is presented in Fig. 1. The KDCA establishes the Infectious Disease Control Committee, a pivotal body charged with deliberating on major policies concerning infectious disease prevention and management.

The committee's responsibilities include formulating basic plans, overseeing the provision of medical care, conducting investigations and research on infectious diseases, disseminating knowledge, issuing autopsy orders, determining vaccination criteria, establishing crisis management measures, and various other aspects integral to combating infectious diseases.

Within this legal framework, the Korea Expert Committee on Immunization Practices (KECIP), as a subcommittee of the Infectious Disease Control Committee, is tasked with making key decisions regarding vaccination policy. This includes identifying infectious diseases that require national immunization, establishing eligibility criteria and methods for vaccination. The KECIP's recommendations are guided by the Ministry of Health and Welfare and directly influence the KDCA's implementation of national vaccination strategies. While KECIP focuses on policy recommendations, other entities within KDCA, such as the Korea Vaccine Injury Compensation Committee, are responsible for vaccine safety investigations and compensation processes. The establishment and functioning of subcommittees and matters beyond the purview of specific articles are determined by presidential decree, ensuring a comprehensive and robust legal framework for infectious disease control and vaccination management in South Korea.

## 4 Deliberation Topics of the KECIP

The Korea Expert Committee on Immunization Practices (KECIP) plays a pivotal role in evaluating and guiding various facets of immunization. The committee's focus encompasses a spectrum of deliberations, [16] including:

Designation or Cancellation of Infectious Diseases for National Immunization Program: The committee engages in discussions to identify or rescind the designation of infectious diseases necessitating vaccination as shown in Table 1. While Table 1 focuses on childhood vaccinations, KECIP's recommendations extend to all age groups, including adults and adolescents, as well as specific high-risk populations such as healthcare workers, the elderly, and immunocompromised individuals. However, mandatory vaccination laws targeting the general population, including children, are absent. Instead, recommendations are issued by governmental bodies and the KECIP, lacking legal enforcement. Nonetheless, these recommendations receive endorsement from academic societies and widespread support from pediatric healthcare providers nationwide. A school entrance check system mandates specific vaccines (MMR, DTaP, IPV, Japanese encephalitis) prior to elementary and middle school entry. Furthermore, broad medical access facilitates easy and cost-free vaccination for all demographics.

**Table 1:** National Immunization Program Status in South Korea.

Type of vaccines	No. of doses	At birth	<4wks	1mo	2mo	4mo	6mo	12mo	15mo	18mo	19-23mo	24-35mo	4 yr	6 yr	11 yr	12 yr
HepB	3	1st		2nd			3rd									
BCG	1		1st													
DTaP	5				1st	2nd	3rd		4th				5th			
Tdap/Td	1														6th	
IPV	4				1st	2nd	3rd						4th			
Hib	4				1st	2nd	3rd	4th								
PCV	4				1st	2nd	3rd	4th								
PPSV	–											High risk population				
RV1	2				1st	2nd										
RV5	3				1st	2nd	3rd									
MMR	2							1st					2nd			
VAR	1							1st								
HepA	2							1st ∼ 2nd								
IJEV	5							1st ∼ 2nd				3rd		4th		5th
LJEV	2							1st				2nd				
HPV	2														1st ∼ 2nd	
IIV	–						Annual vaccination									

Abbreviation. HepB, hepatitis B; BCG, Bacille Calmette-Guérin; DTaP, diphtheria, tetanus, acellular pertussis; Tdap/Td, tetanus, diphtheria, acellular pertussis/tetanus, diphtheria; IPV, inactivated poliovirus vaccine; Hib, *Haemophilus influenzae* type b; PCV, pneumococcal conjugate vaccine; PPSV, pneumococcal polysaccharide vaccine; RV1/RV5, rotavirus vaccines; MMR, measles-mumps-rubella; VAR, varicella; HepA, hepatitis A; IJEV, inactivated Japanese encephalitis; LJEV, live-attenuated Japanese encephalitis; HPV, human papillomavirus; IIV, inactivated influenza vaccine.

Criteria and Methods for Vaccination Implementation: Deliberations center around establishing criteria and methodologies for the execution of vaccination programs.

Policies on the Management of Targeted Infectious Diseases: The committee formulates policies pertaining to the management of infectious diseases specifically targeted for vaccination.

Eradication and Elimination Strategies: Matters related to the eradication and elimination of infectious diseases subject to vaccination are scrutinized to enhance public health outcomes.

Other Immunization-Related Issues: The committee addresses additional issues related to immunization as deemed essential by chairman of the Infectious Diseases Control Committee. For example, KECIP interacts with vaccine manufacturers transparently, requiring members to disclose any financial ties and recusing conflicted members from relevant discussions. While maintaining independence, KECIP may seek industry input on vaccine development, safety, and supply, and request data for informed decision-making. Also, KECIP interacts with the Ministry of Food and Drug Safety Agency (MFDS) in several ways to ensure the safety and efficacy of vaccines. Firstly, KECIP relies on the MFDS' regulatory approval process to assess the safety and efficacy of vaccines before making recommendations. KECIP often reviews MFDS' evaluation of clinical trial data and regulatory decisions as part of its own deliberations. Additionally, KECIP may consult with MFDS experts on matters related to vaccine development, safety monitoring, and regulatory guidance. While KECIP's recommendations inform national immunization practices, a systematic process to ensure their formal alignment with regulatory standards is not currently in place.

During the COVID-19 pandemic, KECIP faced challenges in swiftly evaluating the safety and efficacy of newly developed vaccines, given limited data and uncertainty surrounding their long-term effects. Prioritizing vaccine distribution amidst supply constraints and adapting recommendations in response to emerging variants and evolving evidence were also crucial tasks. Effective communication to address vaccine hesitancy and foster public trust, along with global collaboration to ensure a coordinated response, were imperative.

## 5 Operation and procedures

The committee follows a biannual schedule for regular meetings, with each session typically spanning one day. However, the frequency of meetings can be augmented based on emerging needs and circumstances. Supplementary sessions are commonly necessitated, leading to an annual count exceeding the prescribed two meetings in particular years. Moreover, special committee convocations can be prompted at the request of a majority of members or upon being deemed essential by the chairperson of the Infectious Disease Control Committee or the Commission of the Korea Disease Control and Prevention Agency (KDCA). [16] Operational measures ensuring the committee's efficiency are determined through committee resolutions, guided by the committee chairman. Supplementary operational details are decided through resolutions under the chairman's direction, beyond those explicitly outlined in the regulations.

The committee comprises up to 15 members, including a chairman and a vice-chairman, each serving a two-year term. The chairman is appointed by the chairman of the Infectious Disease Control Committee from within its members, while the vice-chairman is selected from among the KECIP members. Committee members, representing diverse expertise, are appointed or commissioned by the chairman of the Infectious Diseases Control Committee and fall into distinct categories. These include members of the Infectious Diseases Control Committee, senior government officials from the Ministry of Food and Drug Safety (MFDS) and KDCA, clinical physicians experienced in vaccination practices, experts recommended by civic organizations in vaccination, specialists in preventive medicine, public health, infectious diseases targeted for vaccination, immunology, microbiology, and health economics related to vaccination, all recommended by relevant societies, organizations, or the Infectious Diseases Control Committee. The two-year term of office ensures continuity, and appointed members serve throughout their tenure. The inclusion of government officials from the MFDS and KDCA as voting members is a distinctive feature of KECIP, differing from similar committees in some other countries where government representatives often serve in ex-officio roles. This structure fosters a more integrated approach, allowing KECIP to be involved in aspects beyond recommendations, including considerations related to safety monitoring and compensation. While this approach can lead to streamlined decision-making and faster implementation, it also necessitates careful management to maintain objectivity and avoid potential conflicts of interest. The chairperson's discretion to amend operational details provides flexibility, enabling the committee to adapt to evolving circumstances and address unique challenges promptly, though it requires judicious application to ensure consistency and fairness in the committee's proceedings.

The KECIP's operations are facilitated by a dedicated Secretariat, which is integral to the committee's administrative functioning. This Secretariat, embedded within the Immunization Program of the KDCA, provides essential logistical and organizational support, thereby ensuring the committee's operational efficacy.

## 6 Operation of specialized subcommittees

Specialized subcommittees play a crucial role in the intricate framework of the Infectious Diseases Prevention and Management Act, particularly in addressing the nuances of infectious disease control and vaccination. These subcommittees, while primarily composed of Korea Expert Committee on Immunization Practices members, may also include invited external experts when specific expertise is required. They are convened at the discretion of the subcommittee chairman or when a need is identified by the chairman or a majority of subcommittee members. For instance, a specialized subcommittee might be formed to rapidly evaluate and make recommendations for a new vaccine during an emerging infectious disease outbreak, demonstrating their ability to address urgent, situation-specific issues.

Meetings of specialized subcommittees require the participation of a majority of its members for initiation, and decisions are reached through the approval of a majority of the attending members. This ensures that key decisions are made collectively, reflecting the diverse expertise and perspectives within the subcommittee.

The chairman of a specialized subcommittee serves as a vital link between the subcommittee's deliberations and the broader Committee, as they are responsible for reporting the outcomes and resolutions of the subcommittee to the chairman of the overarching committee. This reporting mechanism ensures transparency and coherence in the decision-making process, aligning the efforts of the subcommittee with the broader objectives outlined by the Committee.

To facilitate the efficient operation of specialized subcommittees, additional matters beyond the specifics outlined in relevant articles are determined by the chairman of the specialized subcommittee. Resolutions of the specialized subcommittee provide the mechanism for addressing these operational necessities, with due consideration to any amendments or modifications that may arise in the course of the committee's work. This adaptive approach, amended on January 6, 2015, underscores the dynamic nature of infectious disease control and vaccination strategies, allowing for timely responses to emerging challenges and opportunities within the scope of the legal framework.

## 7 Operational protocols

The Korea Expert Committee on Immunization Practices (KECIP) functions with a well-defined structure, where each member plays a distinct role in the efficient operation of the committee. This section sheds light on the roles, attendance requirements, secretary's responsibilities, and ethical obligations associated with committee members.

The Chairman assumes a pivotal role as the representative of the committee, overseeing its operations, while the Vice Chairman provides essential support and acts as a proxy in the Chairman's absence. The tenure of appointed members corresponds to their respective positions, and commissioned members serve a standard term of two years, subject to reappointment. While there is no strict limit on the number of reappointments, members typically serve one or two additional terms, based on their expertise, contributions, and the committee's needs.

Attendance at meetings is mandatory for all members, with provisions for exceptional circumstances. In the absence of a member due to unavoidable reasons, a designated deputy may attend, speak, or vote on their behalf, provided they possess a delegation letter issued by the absent member. This provision ensures a continuous and informed decision-making process.

The appointment of an executive secretary, who heads the department responsible for vaccination activities, ensures the smooth administrative functioning of the committee. The secretary is tasked with preparing and maintaining minutes, fostering transparency and accountability. These minutes are disclosed through information and communication networks before the committee's subsequent meeting, with the committee having the authority to specify the scope and content of the information to be disclosed.

The Chairman holds the authority to dismiss or remove a member under specified conditions. These conditions include the inability to perform duties due to physical or mental abnormalities, consecutive unexplained absences from meetings, conflicts of interest impairing fair duty execution, lack of expertise essential to committee functions, failure to disclose conflicts of interest, or unauthorized disclosure of deliberation content. This mechanism ensures the integrity and competency of committee members.

The KECIP employs a process to evaluate evidence strength for vaccine policy decisions, involving the review of scientific literature such as randomized controlled trials, observational studies, and post-marketing surveillance data. While KECIP assesses evidence quality, it currently lacks a standardized grading system based on scientific support levels. Decision-making incorporates considerations of benefits, risks, public health impact, and stakeholder input. The committee recognizes the potential benefits of adopting a more systematic approach to evidence evaluation, such as the GRADE framework, to enhance consistency and transparency in its decision-making process. This is an area under active consideration for future improvements in KECIP's operational protocols.

## 8 Ethical obligations

The members of the Korea Expert Committee on Immunization Practices (KECIP) are ethically bound to disclose any economic interests tied to vaccine-related companies for themselves or their families. This obligation is reinforced by the prompt submission of a Conflict of Interest Declaration Form to the secretary by members with economic interests. The KECIP charter and the Conflict of Interest Declaration Form, which members are required to complete annually, provide written standards detailing what constitutes a conflict of interest. Failure to adhere to these obligations renders the member's vote invalid, prompting a reevaluation by an ad-hoc committee if necessary. Further, the Chairman may escalate the matter for dismissal or removal if ethical breaches persist. Accordingly, members with an economic interest are automatically expected to abstain from voting on related vaccine recommendations. This measure ensures our research and recommendations maintain the highest level of objectivity.

In essence, these guidelines and protocols contribute to a robust and ethically sound operation of the KECIP, aligning with the broader objectives of immunization practices and infectious disease management.

## 9 Confidentiality, allowances, and meeting protocols

Maintaining the confidentiality of information garnered during committee proceedings is paramount to ensuring the integrity of the Korea Expert Committee on Immunization Practices (KECIP). Committee members are ethically bound not to disclose or exploit acquired information for unauthorized purposes. The representation of the committee, publicly or otherwise, necessitates prior committee approval. In cases where a member intends to disclose committee-approved information, advanced consultation with the secretary is mandated, with careful consideration to avoid exposing data exclusive to the Korea Disease Control and Prevention Agency (KDCA).

Effective meeting operations are central to the committee's functionality. Regular meeting dates are established during committee sessions, with the chairman providing a minimum seven-day notice, including the agenda, to committee members. Urgent situations or specific circumstances, as outlined in Article 11, Paragraph 1, may warrant deviations from this norm. Meetings are predominantly conducted in-person or via video conferences. However, under specific conditions, ‘written meetings’ - which involve discussions and decision-making via email exchanges - are also permitted. These written meetings document key discussions, decisions, and action items, serving as an official record of the committee's activities. The chairman may invite relevant individuals to offer insights during meetings, with important matters reported to higher authorities for transparency.

Meetings are generally open to the public to foster transparency. Regular biannual sessions of KECIP are typically open, allowing public attendance. In line with KECIP's commitment to transparency and inclusivity, members of the public have the opportunity to contribute to the discussion during meetings, provided they receive prior approval from the chairperson. This approach facilitates a broader engagement and allows the committee to consider diverse viewpoints, enhancing the robustness of its recommendations. However, certain situations may prompt the chairman, in consultation with the secretary, to decide against public disclosure. These include concerns about national security, order maintenance, public welfare, potential damage to individual or organizational reputation, and impediments to fair business operations. Attendance procedures and limitations are in place, ensuring a balanced representation and orderly conduct. Individuals, corporations, or organizations wishing to attend must adhere to a formal application process.

To ensure public representation in the decision-making process, KECIP has included a representative from a consumer group as a committee member since its inception in 1992. This long-standing practice aims to incorporate public opinion into the committee's deliberations and recommendations. While KECIP continues to explore additional means of direct communication with the public, this consumer representative serves as a crucial link between the committee and the general population.

The allocation of funds is crucial for the effective functioning of the committee. The total budget for 2022 is specified, with allowances and expenses permissible within this framework. However, exemptions exist for government officials attending in their official capacities. A review of the recent 3-year budget execution performance, specifically for main meetings, is a key component in assessing the financial efficacy of the committee.

In essence, these measures underscore the commitment of the KECIP to operate with transparency, fiscal responsibility, and adherence to ethical standards, crucial elements in fostering public trust and effective committee functioning.

## 10 Optimizing expertise: functioning of vaccination expert advisory panels

In pursuit of comprehensive insights into vaccination-related matters, the Korea Expert Committee on Immunization Practices (KECIP) can establish Vaccination Expert Advisory Panels, each focused on a specific area. These panels, meticulously curated to cover diverse vaccination realms, are pivotal for in-depth investigations, analyses, and counsel. The spectrum of expert panels spans a multitude of areas, including Tuberculosis, Measles-Mumps-Rubella (MMR), Influenza, and Economic Evaluation, to name a few. The formation of such panels caters to the need for specialized knowledge in steering vaccination strategies. However, final decisions are made by the full KECIP, with meeting frequency varying based on the requirements for expert advice on investigative and analytical matters. All expert panel members complete conflict of interest declarations, ensuring transparency and accountability.

Advisory members for the Vaccination Expert Advisory Panels are meticulously selected by the head of the department overseeing vaccination tasks at the Korea Disease Control and Prevention Agency (KDCA). Drawing from various fields, these experts contribute valuable perspectives. Clinicians with extensive vaccination implementation experience, professionals in vaccine clinical trials, experts in microbiology and virology, and specialists in health economics are among those recommended by relevant societies. The appointment of individuals recognized as indispensable for major committee tasks rests with the Director of KDCA or the Chairman.

**Fig. 2:**
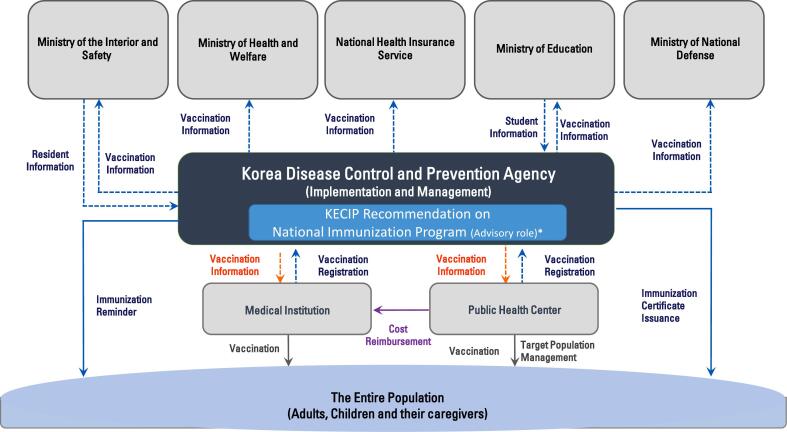
National Immunization Program Coordination and Information Flowchart (Implemented by Korea Disease Control and Prevention Agency based on KECIP recommendations).

The Vaccination Expert Advisory Panels engage in multifaceted advisory activities pertaining to vaccination and targeted infectious diseases. Operating under the chairman's approval, these panels delve into research, guideline development, and expert opinions presented during committee meetings. Their responsibilities encompass investigating the feasibility of national vaccination support projects, assessing the immune response to diseases targeted for vaccination, and planning for the stable supply of national vaccination vaccines. Fig. 2 further shows the intricate work of the Vaccination Expert Advisory Panels by outlining the service flowchart of the immunization integrated management system.

To ensure streamlined operations, a secretary is appointed for each Vaccination Expert Advisory Panel, overseeing tasks within the vaccination domain. In instances requiring separate meetings or involving these panels in committee sessions, ethical obligations, as outlined in Article 9, are binding on expert advisory members. The appointment of a panel chairman may be commissioned to enhance operational efficiency.

## 11 Comparison of KECIP with other National Immunization Technical Advisory Groups.

The Korea Expert Committee on Immunization Practices (KECIP) shares many characteristics with National Immunization Technical Advisory Groups (NITAGs) in other countries, while also maintaining unique features. [[17], [18], [19]] This section provides a comparative analysis of KECIP with NITAGs from the United States (Advisory Committee on Immunization Practices, ACIP), [[20], [21], [22], [23], [24]] United Kingdom (Joint Committee on Vaccination and Immunization, JCVI), [[25], [26], [27]] Australia (Australian Technical Advisory Group on Immunization, ATAGI), [[28], [29], [30]] and Canada (National Advisory Committee on Immunization, NACI). [31,32]

Establishment and Legal Basis: KECIP was established in 1992 and gained legal status in 1994, which is relatively recent compared to ACIP (1964) and JCVI (1963), but predates ATAGI (1997). This timeline reflects the global trend of formalizing immunization advisory bodies in the latter half of the 20th century.

Committee Composition: KECIP comprises up to 15 members, which is comparable to other NITAGs. However, KECIP's structure is simpler, lacking the extensive network of ex-officio and liaison members seen in ACIP or the consumer representatives included in ATAGI.

Meeting Frequency and Transparency: KECIP holds biannual regular meetings, which is less frequent than ACIP (three times per year) or ATAGI (six times per year). However, KECIP maintains a similar level of transparency, with public access to meetings, aligning with global best practices in governance.

Subcommittees and Working Groups: Unlike ACIP, JCVI, ATAGI, and NACI, KECIP does not currently operate standing subcommittees or working groups. This structural difference may impact the depth and breadth of topic-specific reviews and recommendations.

Policy Impact: KECIP's recommendations have a more direct path to implementation through the Korea Disease Control and Prevention Agency, contrasting with the advisory nature of recommendations from ACIP or JCVI, which require additional steps for policy adoption.

Evidence Evaluation: While all NITAGs strive for evidence-based recommendations, KECIP's approach to systematic evidence review and grading (such as the GRADE system used by ACIP) could be further developed to align with international standards.

This comparative analysis highlights areas where KECIP aligns with international NITAG practices and identifies potential areas for enhancement, such as the establishment of subcommittees, increased meeting frequency, and more structured evidence evaluation processes. The detailed comparison of KECIP with other NITAGs is presented in Table 2.

**Table 2:** Comparison of KECIP with other National Immunization Technical Advisory Groups.

Characteristics	Korea (KECIP)	United States (ACIP)	United Kingdom (JCVI)	Australia (ATAGI)	Canada (NACI)
Establishment Year	Established in 1992 (Elevated to legal entity in 1994)	Established in 1964 (Designated as federal advisory committee in 1972)	Established in 1963 (Permanent advisory committee in 1981)	Established in 1997	Established in 1964 (Renamed in 1978)
Legal Basis	Infectious Disease Control and Prevention Act	Public Health Service Act	NHS Act	National Health Act	–
Decision-Making Process	Majority vote of attending members (Majority of registered members for written meetings)	Voting	Consensus based on expert opinions	Voting (Consensus if possible, Chair has casting vote in case of tie)	Voting
Committee Composition	Up to 15 members	15 voting members, 6 ex officio members, 30 non-voting liaison organization representatives	16 members (varies by period)	15 standing voting members, 4 ex officio members	15 voting members, 9 non-voting members, 6 ex officio members
Conflict of Interest Disclosure	Mandatory disclosure (Submission of conflict of interest declaration)	Verification before meetings and voting (Members with vaccine company interests ineligible)	Members with interests may participate in discussions but not decision-making	Decide whether to exclude from meeting or decision-making based on interests	Members with interests excluded from voting
Member Term	Appointed members: 2 years (Nominated members: duration of position)	4 years (Chair: 3 years)	Maximum 3 years (Renewable, not exceeding 10 years)	Standing members: maximum 4 years	Members: 4 years (renewable once), Chair/Vice-Chair: 2 years (maximum 4 years)
Operating Budget	7,000,000 KRW (2022)	$514,560 (including member compensation and travel expenses)	No allocated budget	Funded by Department of Health, partial pharmaceutical company support	–
Meeting Frequency	Biannual regular meetings (ad hoc meetings as needed)	Triannual regular meetings	Triannual meetings (additional meetings in emergencies)	Six regular meetings annually (two via videoconference)	Three face-to-face meetings annually (monthly videoconferences)
Meeting Transparency	Principally open (individuals and corporations may observe)	Webcast (Schedule announced at least 15 days in advance)	Observers (government and agency representatives, not general public)	Meeting dates posted on ATAGI website at least 12 months in advance	Closed
Minutes Publication	Published via information and communication networks	Published on ACIP website within 90 days post-meeting	Published on website within 6 weeks post-meeting	Published on website at least 6 weeks post-meeting	Summary of minutes published
Member Notification	Schedule notified 7 days pre-meeting	Schedule announced at least 15 days pre-meeting	Meeting notice 7 days in advance	Agenda notified at least 4 weeks pre-meeting	Schedule posted on website 12 months in advance, agenda shared pre-meeting
Subcommittee Operation	Not operated	Operated	Operated	Operated	Operated
Subcommittee Role	–	ACIP member support (data collection, analysis, effectiveness review); Monthly remote meetings (minutes confidential)	JCVI member support (literature and vaccine efficacy review); Meetings as needed (minutes published post-JCVI approval)	ATAGI member support (vaccine utility, prioritization, distribution, safety assessment)	NACI member support (draft preparation for discussion/voting)
Additional Notes	Operation of Expert Advisory Group on Immunization (consultation on specialized knowledge, investigation, and analysis)	–	–	Subcommittees operate as 3 subgroups	Subcommittees established as needed for NACI workplan (meetings biweekly)

Abbreviation. KECIP, Korea Expert Committee on Immunization Practices; ACIP, Advisory Committee on Immunization Practices; JCVI, Joint Committee on Vaccination and Immunization; ATAGI, Australian Technical Advisory Group on Immunization; NACI, National Advisory Committee on Immunization.

## 12 Conclusion

The Korea Expert Committee on Immunization Practices (KECIP) played a pivotal role in shaping the planning and trajectory of the national vaccination program. Delving into diverse topics, the committee has engaged in discussions encompassing additional vaccination timing, expanding vaccine coverage across age groups, emergency approval for pediatric vaccines, and foundational vaccinations for infants. In essence, the strategic evolution of vaccination programs, as orchestrated by the KECIP, reflects a harmonious blend of scientific rigor, expert insights, and a commitment to public health, navigating the dynamic landscape of infectious disease control. In conclusion, reflecting on KECIP's role as a National Immunization Technical Advisory Group (NITAG), it is evident that the committee aligns with the core functions outlined by the World Health Organization. KECIP provides independent, evidence-based advice on immunization policies and practices, mirroring its international counterparts. However, KECIP's unique characteristics, such as its close alignment with the Korea Disease Control and Prevention Agency, allow for more direct policy implementation. Moving forward, KECIP could consider adopting practices from other NITAGs to enhance its effectiveness, such as establishing specialized subcommittees and formalizing evidence evaluation processes. By balancing its strengths with lessons from global NITAG practices, KECIP can further strengthen its crucial role in shaping South Korea's immunization policies and contributing to public health outcomes.

## Funding

This work was supported by grants from the Korea Disease Control and Prevention Agency (grant no. 2023–10-019).

## CRediT authorship contribution statement

**So Yun Lim:** Writing – original draft, Visualization, Investigation, Formal analysis, Data curation. **Han Wool Kim:** Writing – review & editing, Investigation, Formal analysis, Data curation. **Young June Choe:** Writing – original draft, Visualization, Validation, Supervision, Methodology, Formal analysis, Conceptualization. **Bin Ahn:** Writing – review & editing, Investigation, Data curation. **Hyun Mi Kang:** Writing – review & editing, Investigation, Data curation. **Jiyoung Park:** Writing – review & editing, Investigation, Data curation. **Geun Yong Kwon:** Writing – review & editing, Resources, Project administration, Conceptualization. **Seung Ho Lee:** Writing – review & editing, Supervision, Resources, Project administration. **Sunghee Kwon:** Writing – review & editing, Visualization, Project administration, Methodology. **Eun Hwa Choi:** Writing – review & editing, Supervision, Funding acquisition, Conceptualization.

## Declaration of competing interest

The authors declare that they have no known competing financial interests or personal relationships that could have appeared to influence the work reported in this paper.

## Data Availability

Data will be made available on request.
